# Muscle Wasting and Impaired Myogenesis in Tumor Bearing Mice Are Prevented by ERK Inhibition

**DOI:** 10.1371/journal.pone.0013604

**Published:** 2010-10-27

**Authors:** Fabio Penna, Domiziana Costamagna, Alessandro Fanzani, Gabriella Bonelli, Francesco M. Baccino, Paola Costelli

**Affiliations:** 1 Department of Experimental Medicine and Oncology, University of Torino, Torino, Italy; 2 Department of Biomedical Sciences and Biotechnology, University of Brescia, Brescia, Italy; McMaster University, Canada

## Abstract

**Background:**

The onset of cachexia is a frequent feature in cancer patients. Prominent characteristic of this syndrome is the loss of body and muscle weight, this latter being mainly supported by increased protein breakdown rates. While the signaling pathways dependent on IGF-1 or myostatin were causally involved in muscle atrophy, the role of the Mitogen-Activated-Protein-Kinases is still largely debated. The present study investigated this point on mice bearing the C26 colon adenocarcinoma.

**Methodology/Principal Findings:**

C26-bearing mice display a marked loss of body weight and muscle mass, this latter associated with increased phosphorylated (p)-ERK. Administration of the ERK inhibitor PD98059 to tumor bearers attenuates muscle depletion and weakness, while restoring normal atrogin-1 expression. In C26 hosts, muscle wasting is also associated with increased Pax7 expression and reduced myogenin levels. Such pattern, suggestive of impaired myogenesis, is reversed by PD98059. Increased p-ERK and reduced myosin heavy chain content can be observed in TNFα-treated C2C12 myotubes, while decreased myogenin and MyoD levels occur in differentiating myoblasts exposed to the cytokine. All these changes are prevented by PD98059.

**Conclusions/Significance:**

These results demonstrate that ERK is involved in the pathogenesis of muscle wasting in cancer cachexia and could thus be proposed as a therapeutic target.

## Introduction

Cancer cachexia is a multifactorial, multifaceted syndrome, in particular characterized by marked loss of body weight, depletion of fat and muscle mass and protein hypercatabolism in many tissues. The onset of cachexia is clinically relevant since it complicates patients' management by both increasing morbidity and mortality rates and reducing the tolerance to antineoplastic treatments. Anorexia, inflammation and altered hormonal homeostasis significantly contribute to the pathogenesis of cancer cachexia.

Skeletal muscle wasting is a major feature of cachexia. Among the underlying mechanisms, a prominent role is played by the onset of a sustained hypercatabolic response not directly related to the tumor-host metabolic needs. The enhanced muscle protein degradation relies on the activity of different proteolytic systems. The ubiquitin and proteasome system (UPS), in particular, seems to play a major role [1], although additional proteolytic pathways were proposed to act upstream and downstream of the UPS to accomplish complete breakdown of myofibrillar proteins [2]. Finally, a role for the autophagic-lysosomal degradation has been revisited [3]. Other mechanisms possibly accounting for muscle depletion in cancer cachexia are downregulation of protein synthesis rates[4], or an impaired myogenic regenerative response [5].

Recently, specific signaling pathways have been suggested to contribute to muscle atrophy. In this regard, downregulated IGF1 signaling was associated with muscle atrophy by denervation, immobilization, unloading, starvation, aging, and prolonged glucocorticoid administration [6]. By contrast, the activation status of the IGF1-dependent signaling was not impaired in the skeletal muscle of tumor-bearing animals [7]. This observation led to search for alternative signaling pathways that might be relevant to the pathogenesis of muscle depletion in experimental cancer cachexia. Keeping in mind that proinflammatory factors play a pivotal role in mediating muscle wasting, the attention was focused on cytokine-dependent signals, in particular those activated by Mitogen-Activated Protein Kinases (MAPKs).

Four main MAPKs have been identified in mammals: JNK (1–3), p38 (α-δ), ERK 1/2 (hereafter referred to as ERK) and ERK5 [8]. MAPKs are activated by phosphorylation of both threonine and tyrosine residues by MAPK-kinases and inactivated by specific phosphatases such as the MAPK-phosphatase 1 [8]. Both MAPKs and phosphatases participate in the regulation of muscle growth and function [9]. p38 activation is necessary for muscle development which leads to phosphorylation of substrates involved in the myogenic process. In particular, p38 was shown to affect the expression of myogenic regulatory factors (MRFs), such as Myf5, and the activities of transcription factors belonging to the MEF2 and MyoD families. In addition, p38 was demonstrated to inhibit ERK, thereby withdrawing myocytes from the cell cycle, while ERK inhibition resulted in marked induction of p38 activity [9]. The interaction between p38 and ERK was proposed to drive the p38-dependent growth arrest and myogenic differentiation in rhabdomyosarcoma cells [10]. In a similar way, ERK activation was shown to inhibit myotube formation, repressing skeletal myogenesis [11]. A recent study reported that ERK is more active in fast- than in slow-twitch muscles [12], suggesting that this activity could be necessary to maintain the fast-twitch phenotype. Subsequently, the same group showed that inhibition of MAPK signaling is associated with reduced expression of fast fiber-specific genes and with a shift towards the slow-twitch fiber phenotype [13]. These observations are in contrast with a previous report showing that in the regenerating soleus muscle the activity of the Ras-ERK pathway is needed to support the production of slow myosin heavy chain (MyHC) [14].

The present study aimed to investigate the involvement of MAPKs in the pathogenesis of muscle depletion in mice bearing the C26 carcinoma. Transplantation of the C26 tumor to a host mouse causes a significant loss of body weight and muscle mass and closely reproduces the clinical features of cancer-induced muscle wasting [7], [15]. The results reported in the present paper point to ERK as a critical contributor to muscle atrophy in experimental cancer cachexia.

## Materials and Methods

All materials were supplied by Sigma (St. Louis, MO, USA), unless differently specified.

### Ethics statement

Experimental animals were cared for in compliance with the Italian Ministry of Health Guidelines (n° 86609 EEC, permit number 106/2007-B) and the *Policy on Humane Care and Use of Laboratory Animals* (NIH 1996). The experimental protocol was approved by the Bioethical Committee of the University of Torino.

### Animals and experimental design

Male Wistar rats weighing about 150 g and Balb-c mice weighing about 20 g were obtained from Charles River Laboratories, Inc. (Calco, LC, Italy) and maintained on a regular dark-light cycle (light from 08:00 to 20:00), with free access to food (Piccioni, Brescia, Italy) and water during the whole experimental period.

Tumor-bearing rats (n = 8) received an intraperitoneal inoculum of Yoshida AH-130 ascites hepatoma cells (∼10^8^ cells/rat), while tumor-bearing mice (n = 8) were inoculated s.c. dorsally with 5×10^5^ C26 undifferentiated carcinoma cells. Healthy rats or mice inoculated with vehicle (saline) served as controls (n = 6).

In another set of experiments, animals were randomized and divided into four groups, namely controls (C, n = 6) and tumor bearers (TB, n = 8), treated or not with the MEK inhibitor PD98059 (PD). PD groups received daily s.c. injections of PD98059 (1 or 3 mg/kg, Calbiochem, La Jolla, CA, USA). In particular, rats bearing the AH-130 tumor received 3 mg/kg PD98059. As for the C26 hosts, two different experiments were performed, treating the animals with 1 or 3 mg/kg PD98059. Untreated animals received an equal amount (100 µl) of vehicle (saline containing 2% DMSO).

Fore and hindlimb grasping strength was measured by means of a grip strength meter (Columbus Instruments, Columbus, OH, USA).

Animal weight and food intake were recorded daily. Tumor-bearing rats and mice were sacrificed under anesthesia 4 and 13 days after tumor transplantation, respectively. Several muscles were rapidly excised, weighed, frozen in isopentane cooled with liquid nitrogen and stored at −80°C.

### Cell cultures

Murine C2C12 skeletal myoblasts (ATCC, Manassas, VA, USA) were grown in high glucose Dulbecco's Modified Eagle's Medium (DMEM) supplemented with 10% FBS, 100 U/ml penicillin, 100 mg/ml streptomycin, 100 mg/ml sodium pyruvate, 2 mM L-glutamine, and maintained at 37°C in a humidified atmosphere of 5% CO_2_ in air. Differentiation was induced by shifting sub-confluent cultures to DMEM supplemented with 2% horse serum (differentiation medium; DM). The medium was changed every 2nd day. On day 0 or 5 of differentiation, the cells were exposed to TNFα (100 ng/ml; Immunological Sciences, Rome, Italy) or to 20 µM PD98059, or both, and collected after 48 h; untreated cells served as control.

### Plasmids and transfection

The pBabe-Puro Myc-Follistatin plasmid (kindly provided by Vittorio Sartorelli, NIH, Bethesda, USA) was purified with a NucleoBond Xtra Maxi kit (Macherey-Nagel GmbH, Duren, Germany). Transfection was performed with the GeneJuice reagent (EMD Biosciences, Madison, WI, USA) following manufacturer instructions. Transfected cells were selected by adding puromycin at the final concentration of 1 µg/ml.

### Immunofluorescence

Transverse sections (10 µm) from the midbelly region of the tibialis anterior muscle were cut on a Leitz 1720 cryostat (Leica, Wetzlar, Germany) and fixed in 4% paraformaldehyde. C2C12 monolayers were washed with PBS and fixed in acetone-methanol (1∶1). Samples were then probed with the following primary antibodies: Laminin from Sigma (St. Louis, MO, USA), Pax7 from the Hybridoma Bank (University of Iowa) and caveolin-1 from Santa Cruz Biotechnology (Santa Cruz, CA, USA). Detection was performed using a Cy3-conjugated mouse IgG secondary antibody (GE Healthcare, Milano, Italy) or a FITC-conjugated rabbit IgG secondary antibody. Nuclei were stained with the DAPI fluorochrome and the images captured in an epiilluminated fluorescence microscope (Axiovert 35, Zeiss, Germany).

### ELISA

IL-6 serum levels were detected by a commercially available mouse ELISA kit, used according to the manufacturer instructions (Bender MedSystems, Vienna, Austria). Serum from each animal (50 µl) was assayed in duplicate. Quantitative calibration was obtained performing a standard curve with recombinant mouse IL-6.

### Western blotting

About 50 mg of muscle were homogenized in 80 mM Tris-HCl, pH 6.8, containing 100 mM DTT, 70 mM SDS, and 1 mM glycerol, with freshly added protease and phosphatase inhibitor cocktails, kept on ice for 30 min, centrifuged at 15000 x g for 10 min at 4°C, and the supernatant collected. Protein concentration was assayed using BSA as working standard. C2C12 cells were lysed on RIPA buffer (50 mM Tris-HCl pH 7.4, 150 mM NaCl, 1% NP40, 0.25% Na-deoxycholate, 1 mM PMSF) with freshly added protease and phosphatase inhibitor cocktails. Equal amounts of protein (30 µg) were heat-denaturated in sample-loading buffer (50 mM Tris-HCl, pH 6.8, 100 mM DTT, 2% SDS, 0.1% bromophenol blue, 10% glycerol), resolved by SDS-PAGE and transferred to nitrocellulose membranes (Bio-Rad, Hercules, CA, USA). The filters were blocked with Tris-buffered saline (TBS) containing 0.05% Tween and 5% non-fat dry milk and then incubated overnight with antibodies directed against: p-ERK (Tyr^204^), ERK, JNK, p-p38 (Tyr^182^), follistatin, MyoD, and myogenin (Santa Cruz Biotechnology, CA, USA), p-JNK (Thr^183^/Tyr^185^), p-c-Jun (Ser^73^) and p-Akt (Ser^473^) (Cell Signaling Technology, Danvers, MA, USA), p38 (Calbiochem, La Jolla, CA, USA), MyHC and tubulin (Sigma, St. Louis, MO, USA), atrogin-1 (ECMbiosciences, Versailles, KY, USA) and the monoclonal antibody against Pax7 developed by Atsushi Kawakami, obtained from the Developmental Studies Hybridoma Bank (University of Iowa). Peroxidase-conjugated IgG (Bio-Rad, Hercules, CA, USA) was used as secondary antibodies. Membrane-bound immune complexes were detected by an enhanced chemiluminescence system (Santa Cruz Biotechnology, USA) on a photon-sensitive film (Hyperfilm ECL, GE Healthcare, Milano, Italy). Protein loading was normalized according to tubulin expression. Specificity of each antibody was tested with positive and negative controls. Quantification of the bands was performed by densitometric analysis using a specific software (TotalLab, NonLinear Dynamics, Newcastle upon Tyne, UK).

### Data analysis and presentation

All results were expressed as mean ± SD. Representative western blots show independent samples. Significance of the differences was evaluated by analysis of variance (ANOVA) followed by Tukey's test.

## Results

Tumor growth in rats bearing the AH-130 hepatoma or mice bearing the C26 carcinoma is associated with progressive loss of body and skeletal muscle wet weight. Activation of a sustained protein hypercatabolic response mainly accounts for muscle depletion [7].

**Figure 1:**
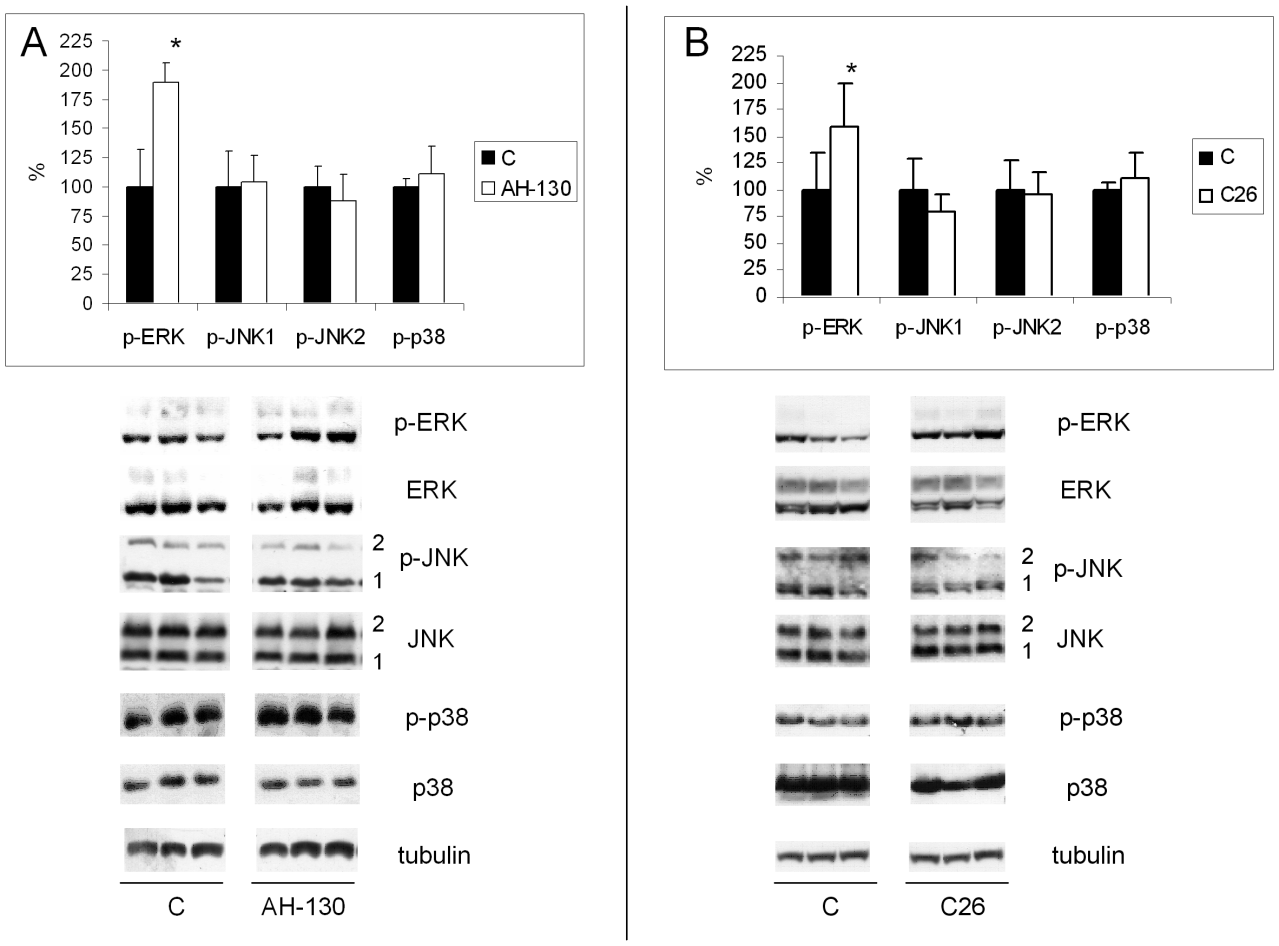
ERK is activated in the skeletal muscle of TB animals. Levels of phosphorylated MAPKs in the GSN. *Panel A*: control (C; n = 6) and AH-130-bearing rats (n = 8). *Panel B*: control (C, n = 8) and C26-bearing mice (n = 8). Levels of phosphorylation were normalized by total protein content. Data (means ± SD) expressed as percentages of controls. Significance of the differences: *p<0.05 vs C.

MAPK expression and phosphorylation status, this latter widely considered a reliable indicator of MAPK activation [8], were evaluated in the gastrocnemius muscle of two experimental models of cancer cachexia. Marked phosphorylation of ERK occurs both in tumor-bearing (TB) rats and mice, reaching levels respectively 90% and 50% higher than in controls. In contrast, JNK and p38 phosphorylation does not change (Fig. 1A, 1B).

### ERK inhibition prevents muscle wasting in the C26 hosts

**Figure 2:**
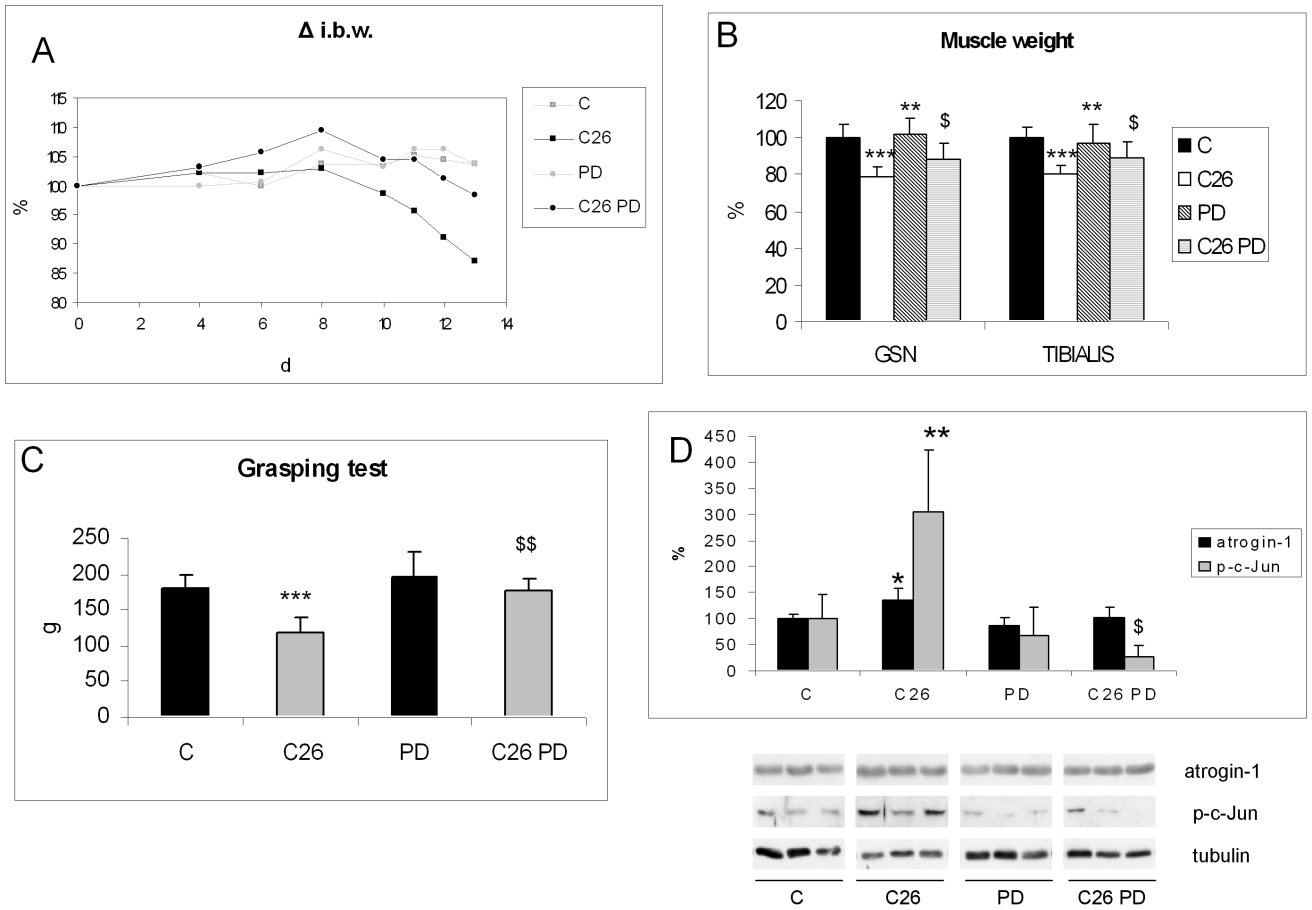
PD98059 counteracts the onset of cachexia in C26-bearing mice. (A) Body weight, expressed as percent changes respect to initial body weight (i.b.w., C = 19.14±1.68 g; C26 = 19.14±0.69 g; PD = 18.57±2.37 g; C26 PD = 17.56±0.89 g), (B) muscle weight, (C) voluntary strength (grasping test), (D) atrogin-1 and p-c-Jun protein expression in the GSN of controls (n = 6) and C26 hosts (n = 8) either untreated or administered PD (1 mg/kg; see Materials and methods). Densitometric quantifications were normalized according to tubulin levels. Data (means ± SD) expressed as percentages of controls. Significance of the differences: *p<0.05 vs C; **p<0.01 vs C; ***p<0.001 vs C; $ p<0.05 vs C26; $$ p<0.01 vs C26.

To assess the relevance of ERK activation to muscle wasting, TB mice were treated with the MEK inhibitor PD98059 (PD) [16]. As Fig. 2A shows, daily PD administration (1 mg/kg) to C26 hosts significantly prevents body weight loss (body weight on day 13: C = 19.8±1.5 g; C26 = 16.7±0.9 g, p *vs* C = 0.0005; PD = 19.7±1.8 g; C26-PD = 18.3±1.6 g, p *vs* C = 0.018, p *vs* C26 = 0.032; n indicated in figure legend), though not affecting tumor growth (C26 = 249±57 mg; C26-PD = 237±31 mg). Moreover, treatment with PD effectively improves the depletion of gastrocnemius and tibialis anterior (Fig. 2B) as well as the muscle grip strength (Fig. 2C). When PD is administered at a dosage of 3 mg/kg, which closely reproduces the concentration adopted by the intramuscular infusion study from Haddad et al. [17], the protective effects reported above are not potentiated any further (Fig. S1). PD-administered AH-130 bearers (3 mg/kg for 4 days) display a pattern comparable to that observed in the C26 hosts receiving the inhibitor (Fig. S2).

Cachexia induced by the C26 tumor appears to depend on elevated interleukin (IL)-6 plasma levels [18]. The present work confirms that circulating IL-6 markedly increases in the C26 hosts, yet PD administration does not prevent this increase (Fig. S3).

To ascertain if PD prevents muscle wasting by downregulating the UPS, protein levels of atrogin-1/MAFbx, a muscle-specific ubiquitin ligase overexpressed in pathological states associated with muscle atrophy [4], [7], were evaluated. Atrogin-1 expression in the gastrocnemius of TB mice is 35% higher than in controls and is slightly reduced by PD treatment (Fig. 2D).

### PD98059 inhibits TNFα-induced myofibrillar protein loss in C2C12 myotubes

**Figure 3:**
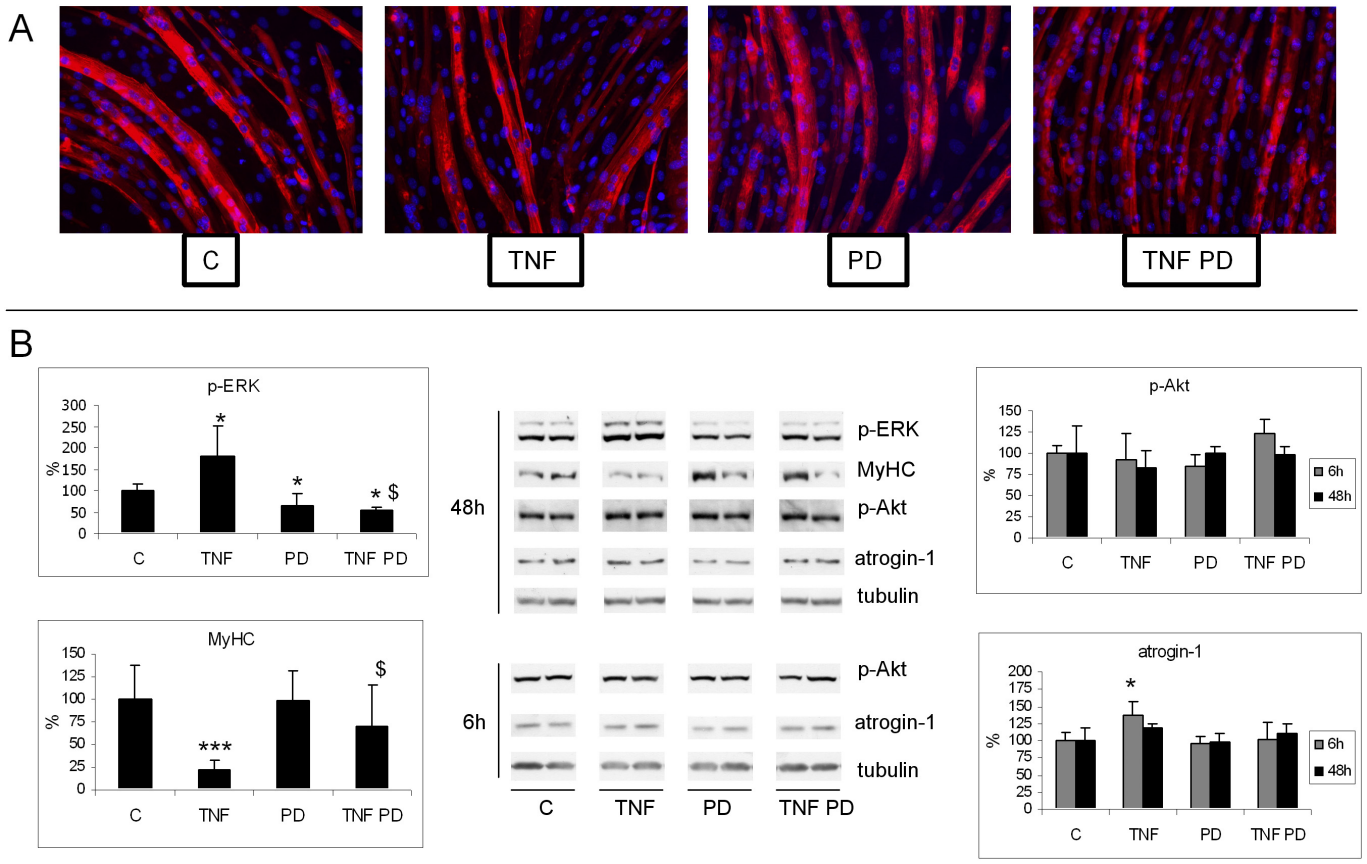
ERK inhibition prevents TNFα-induced alterations in C2C12 myotube cultures. C2C12 myotubes (5 days differentiation) treated for 48 h with 100 ng/ml TNFα, in the presence or in the absence of PD (20μM). (A) MyHC immunostaining (red: MyHC; blue: nuclei). (B) Protein expression levels of p-ERK, MyHC, p-Akt and atrogin-1, the latter two evaluated at both 6 and 48 h (see Li et al., 2005). Densitometric quantifications were normalized according to tubulin levels. Data (means ± SD; n = 3) expressed as percentages of controls. Significance of the differences: *p<0.05 vs C; $ p<0.05 vs TNFα.

To investigate if the proinflammatory cytokine TNFα modulates ERK activation in muscle cells, C2C12 myotubes (5 days in DM) were treated with TNFα for 48 h. As a result, p-ERK levels increase, while both myotube size and MyHC content are reduced. Such changes are significantly inhibited by treatment with PD (Fig. 3A-B). TNFα-induced loss of MyHC is not associated with reduced p-Akt levels, that remain close to control values in cultures either treated or not with PD. Finally, the early (6 h) increase of atrogin-1 expression caused by TNFα is only slightly, but not significantly, attenuated by PD treatment (Fig. 3B).

### ERK activation in TNFα-treated C2C12 myotubes does not depend on myostatin

**Figure 4:**
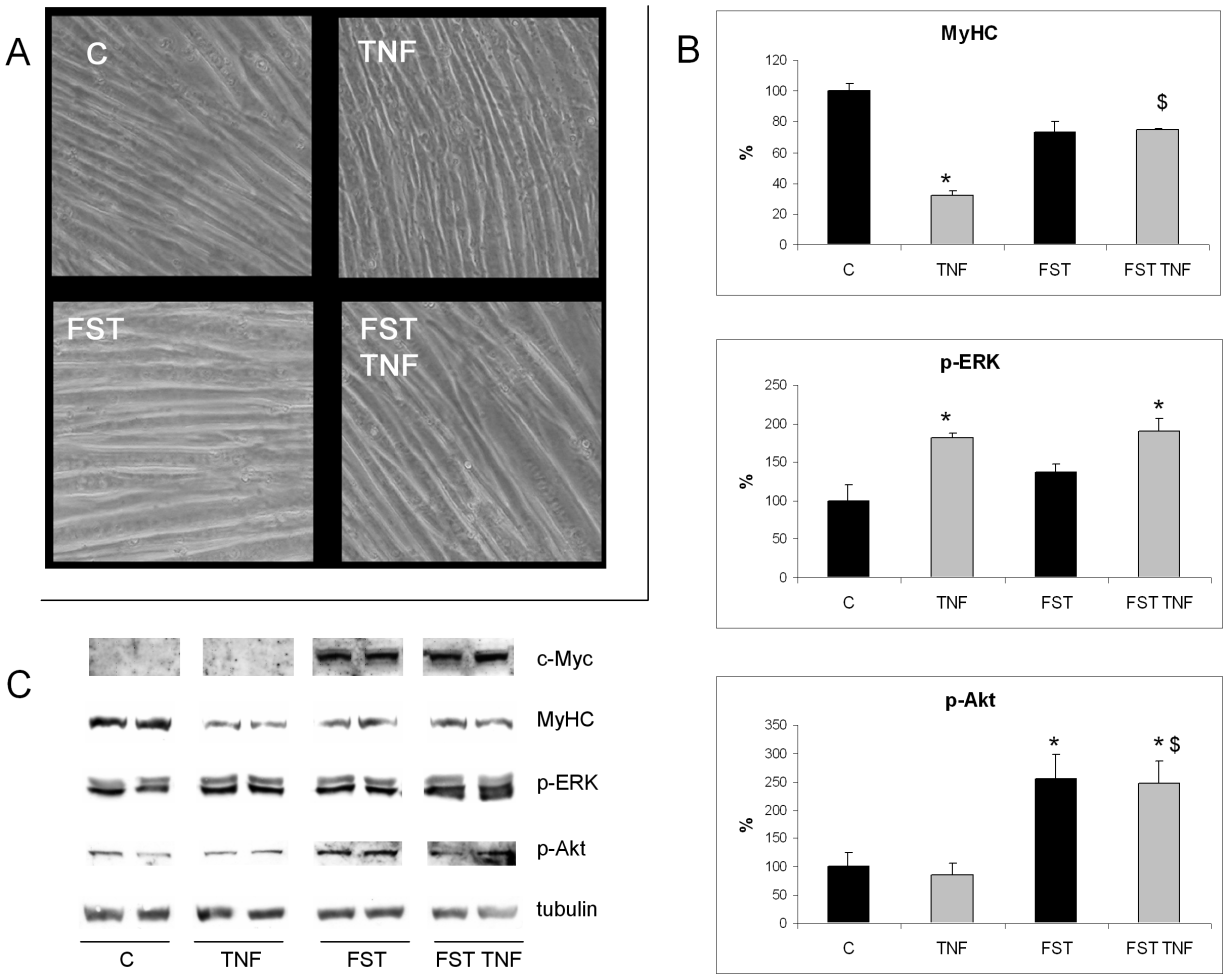
Follistatin overexpression prevents TNFα-induced MyHC loss without interfering with ERK activation. C2C12 myotubes (5 days differentiation), transfected or not with myc-follistatin (FST) and treated or not with 100 ng/ml TNFα for 48 h. (A) Phase contrast microscopy showing the hypertrophy induced by FST in cells treated or not with TNFα. (B) Western blots for MyHC, p-ERK and p-Akt protein levels and (C) corresponding densitometric analysis normalized by tubulin. Data (means ± SD; n = 3) expressed as percentage of controls. Significance of the differences: *p<0.05 vs C; $ p<0.05 vs TNFα.

To verify if TNFα-induced ERK activation might relate to myostatin, C2C12 myoblasts were stably transfected with a vector coding for the endogenous myostatin inhibitor follistatin. No reduction in size or MyHC content occurs in follistatin-overexpressing myotubes treated with TNFα (Fig. 4A-B), although in the latter the degree of ERK activation is similar to that in TNFα-treated non-transfected cells (Fig 4B). On the other hand, follistatin overexpression resulted in increased p-Akt levels, irrespective of the presence of TNFα in the culture medium (Fig. 4B).

### PD98059 prevents satellite cell accumulation in the muscle of C26 hosts

**Figure 5:**
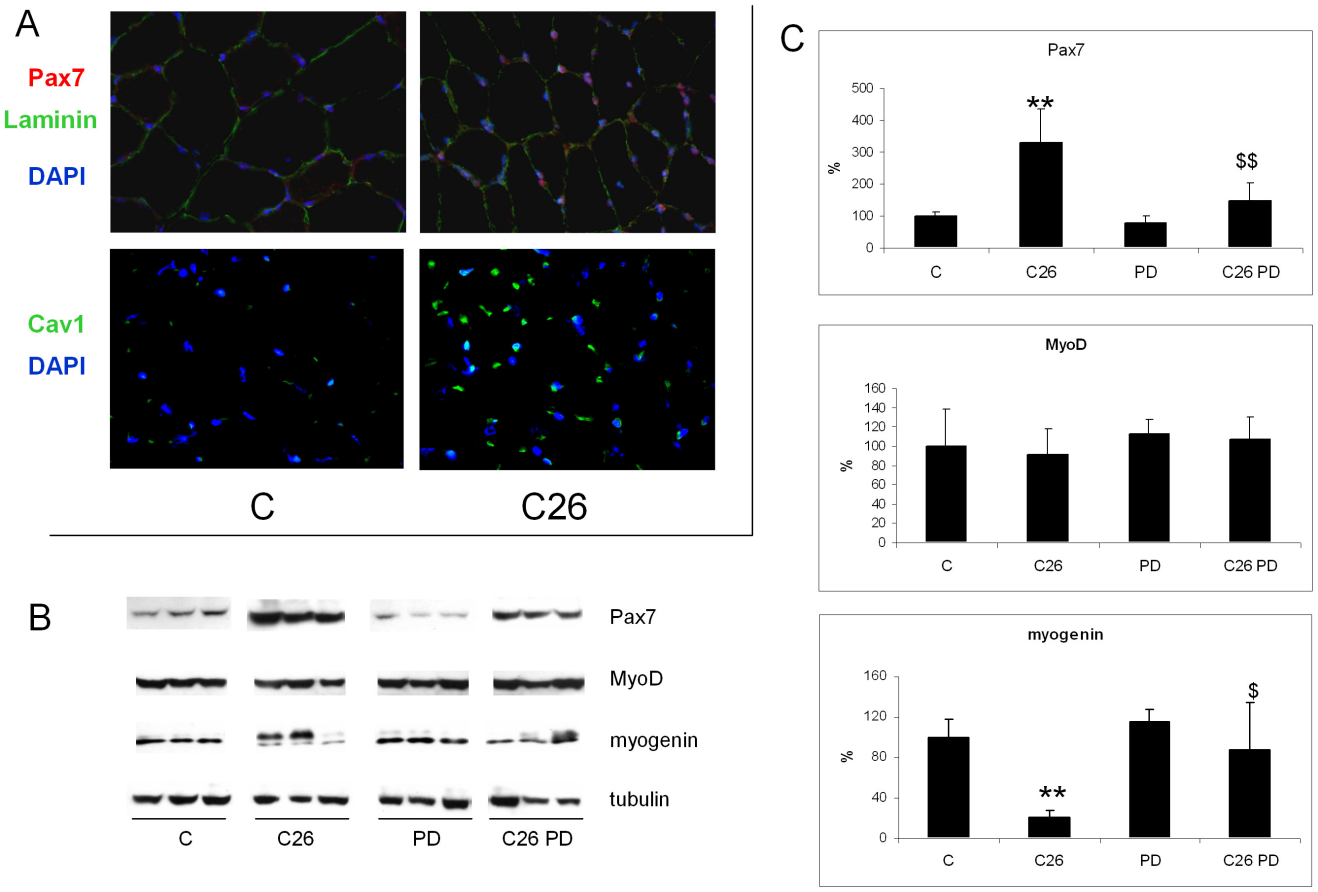
ERK inhibition restores the myogenic potential in TB mice. (A) Immunostaining of Pax7, laminin and caveolin1 (Cav1) in tibialis transverse sections of controls and TB mice. (B) Pax7, MyoD and myogenin protein expression in the GSN of controls (n = 6) and TB mice (n = 8) either untreated or administered PD (1 mg/kg) and (C) corresponding densitometric analysis normalized by tubulin. Data (means ± SD) are expressed as percentages of controls. Significance of the differences: **p<0.01 vs C; $ p<0.05 vs C26; $$ p<0.01 vs C26.

Apart from protein hypercatabolism, muscle wasting could also arise from alterations in the myogenic process. In this regard, aging or hindlimb suspension-induced muscle atrophy are associated with loss of myofiber precursor cells [19]. The present report suggest for the first time that impaired myogenesis significantly contributes to the onset of muscle wasting in cancer cachexia. In the tibialis anterior of the C26 hosts we indeed detected by immunofluorescence microscopy increased levels of Pax7 and caveolin-1 (Fig. 5A), two markers of undifferentiated cells. Pax7 accumulation, also confirmed by western blotting, is largely prevented by PD administration (Fig. 5B).

MyoD levels, previously reported to be reduced in the muscle of AH-130 bearers [20], are unaffected in the gastrocnemius of the C26 hosts (Fig. 5B), while the marked decrease of myogenin is rescued by PD treatment (Fig. 5B). Of interest, two myogenin isoforms can be observed in the TB muscles (Fig. 5B). The one characterized by a slightly higher molecular weight virtually disappears in the PD-treated groups (Fig. 5B) as well as in PD-untreated TB muscles when cell lysates are incubated in the presence of phosphatase (Fig. S4). These observations point to myogenin phosphorylation as a putative mechanism for impaired muscle regenerative response in cancer cachexia. In this regard, phosphorylation in the myogenin transcriptional domain was shown to inhibit the induction of muscle specific genes [21].

### ERK inhibition rescues C2C12 myoblasts from TNFα-induced impaired differentiation

**Figure 6:**
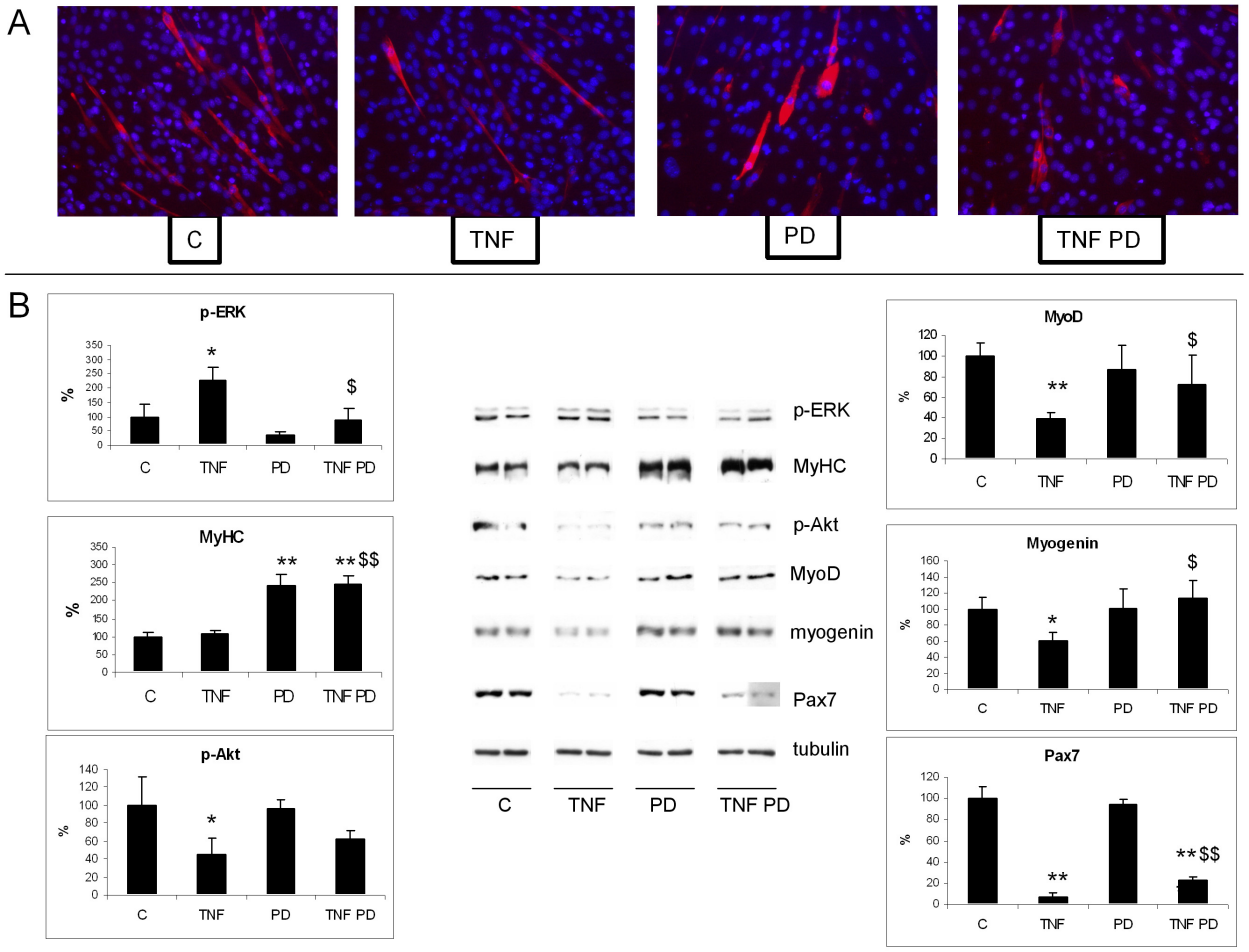
ERK inhibition promotes myogenic differentiation of C2C12 cells. Subconfluent C2C12 cells switched to DM and simultaneously treated for 48 h with 100 ng/ml TNFα in the presence or in the absence of PD (20μM). (A) MyHC immunostaining. (B) Protein expression levels of p-ERK, MyHC, p-Akt, MyoD, myogenin and Pax7. Densitometric quantifications were normalized according to tubulin levels. Data (means ± SD; n = 3) expressed as percentages of controls. Significance of the differences: *p<0.05 vs C; **p<0.01 vs C; $ p<0.05 vs TNFα; $$ p<0.01 vs TNFα.

To better appreciate the role of ERK activation in the regulation of the myogenic program, C2C12 myocytes were exposed to TNFα and PD during the first 48 h in differentiation medium. TNFα enhances ERK phosphoryation, while PD suppresses this effect (Fig. 6B). PD treatment increases MyHC accumulation independently of the presence of TNFα, as shown by both immunofluorescence microscopy and western blotting (Fig. 6A-B). Differently from fully differentiated cells (see Fig. 3B), exposure to the cytokine significantly reduces p-Akt levels and this change is only marginally affected by PD (Fig. 6B). MyoD and myogenin markedly decrease after TNFα exposure and Pax7 expression is virtually abrogated, yet in the presence of PD both MyoD and myogenin maintain control levels, while rescue of Pax7 depletion is only partial (Fig. 6B).

## Discussion

The present study provides the first report that enhanced activity of the MAPK ERK occurs in wasting muscles in the course of experimental cancer cachexia, resulting in atrogin-1 hyperexpression and likely in reduced regenerative potential. Importantly, pharmacological inhibition of ERK in C26 carcinoma-bearing mice markedly attenuates muscle depletion and improves muscle function, without modifying tumor growth or IL-6 circulating levels. This latter observation suggests that the effects observed at the muscle level might not depend on systemic IL-6.

### ERK involvement in muscle wasting

The role of ERK in the pathogenesis of skeletal muscle atrophy is quite controversial. Indeed, ERK inactivation resulted in rat soleus and gastrocnemius atrophy [22] and inhibited fast muscle hypertrophy induced in experimental animals by β_2_-adrenergic agonists or IGF-1 [12], [17]. In addition, reduced levels of phosphorylated ERK were demonstrated during sarcopenia of aging [23]. A recent study shows that ERK activity significantly contributes to the protective effect exerted by IGF-1 against oxidative stress-induced damage in both C2C12 and L6 myocytes [24]. The myogenic regenerative capacity of human satellite cells isolated from both young and old subjects was significantly enhanced when ERK is activated by the presence of FGF in the culture medium. Consistently, both young and old satellite cells failed to produce proliferating fusion-competent myoblasts when ERK was pharmacologically inhibited [23]. Finally, even if not sufficient to lead to myogenesis or muscle hypertrophy, ERK activation regulates satellite cell proliferation, likely contributing to muscle regeneration and hypertrophy [14].

By contrast, increased ERK activation was reported in muscle atrophy induced by hind-limb suspension [25]. In C2C12 myotubes ERK activation resulted in reduced myotube size [16], while ERK inhibition induced a hypertrophic phenotype similar to that elicited by IGF-1 [16]. Similarly, C2C12 myotube cultures subjected to 3D-clinorotation showed an overexpression of both atrogin-1 and MuRF1 ubiquitin ligases, associated with increased levels of phosphorylated ERK [26].

Muscle wasting mainly depends on protein hypercatabolism in which the UPS seems to play a major role [1]. Enhanced UPS-dependent protein breakdown has been associated with downregulation of the IGF1-dependent PI3K/Akt pathway [6], yet the latter is not reduced in the skeletal muscle of tumor-bearing animals [7]. Besides the PI3K/Akt/FoxO signaling, however, other pathways can trigger atrogenes transcription [27]. In this regard, still unclear is the relevance of ERK to the induction of ubiquitin ligases and of the protein hypercatabolic state. In C2C12 myotubes, ERK inhibition appeared required to stimulate ubiquitin ligase expression [13]. Finally, ubiquitin hyperexpression induced by glucocorticoids in L6 myotubes was shown to depend on the activity of both MEK, the kinase upstream of ERK, and the Sp1 transcription factor [28].

### Relevance of TNFα and myostatin to ERK activation

ERK can be activated in the skeletal muscle by several factors, such as cytokines, myostatin, or IGF-1. As for the latter, its contribution to muscle atrophy in cancer cachexia has been already discussed [7]. In the present study we investigated if the proinflammatory cytokine TNFα activates ERK in C2C12 myotube cultures, and whether myostatin is involved in such activation. In this regard, muscle wasting in experimental cancer cachexia was recently suggested to be associated with myostatin upregulation [29], [30]. Similarly, the size reduction caused by TNFα in cultured C2C12 myotubes is accompanied by increased myostatin expression [31]. Finally, myostatin was proposed to activate ERK and to repress the differentiation program in differentiating C2C12 myocytes [32]. In the present study, we show that TNFα caused a marked reduction in myotube size associated with early increased atrogin-1 expression, calpain hyperactivation (C = 1.21*10^−3^±2.3*10^−5^ nkat/mg protein, TNFα = 2.01*10^−3^±2.7*10^−5^ nkat/mg protein, n = 6, p<0.001; see also [33]), and reduced MyHC content, likely due to enhanced myofibrillar protein breakdown. ERK inhibition prevented TNFα-induced changes of myotube size, MyHC content and calpain activity, with a mechanism independent from Akt. This finding is of particular interest in that confirming that atrogin-1 expression and protein breakdown are not regulated by the PI3K/Akt pathway only [6]. The pattern induced by TNFα in C2C12 myotubes closely resembles that observed in the C26 tumor-bearing mice and points to ERK signaling as to another pathway by which proinflammatory cytokines may induce muscle wasting. In this regard, the observations that ERK inhibition in TNFα-treated C2C12 myotubes does not result in modulations of p-Akt, and that ERK is activated in the muscle of the C26 hosts in the absence of any downregulation of p-Akt are intriguing. In fact, although a reciprocal regulation between ERK and Akt has been reported [16], [34], it has not been clearly established in skeletal muscle. In this regard, treatment with PD98059 increases p70S6K activity in L6 myocytes, suggesting that ERK activity results in PI3K/Akt pathway inhibition [35]. By contrast, in the stretched diaphragm muscle, increased p-ERK is dependent on PI3K activity [36], and supplementing serum-starved myoblasts with β-hydroxy-β-methylbutyrate results in increased proliferation and reduced apoptosis due to enhanced phosphorylation of both ERK and Akt [37]. Finally, muscle atrophy induced by conditional activation of Met is associated with increased Akt activation that, at least in isolated myotubes, is paralleled by high levels of p-ERK [38]. Along with other observations showing that biochemical and molecular alterations in the skeletal muscle of cancer patients are detectable even before any evidence of body weight loss [39], these findings suggest that, depending on the situation, the interplay between ERK and Akt might be crucial to preserve muscle mass or to drive the muscle towards atrophy. On this line, ERK activation could be viewed as a compensatory mechanism aimed at increasing satellite cell proliferation.

Myostatin was shown to activate the ERK-dependent pathway [32]. Consistently, ERK activation is associated with myostatin upregulation both in the muscle of tumor-bearing animals [15], [29] and in TNFα-treated C2C12 myotubes[31], suggesting the possibility of a causal relationship between the two events. The results shown in the present study, however, render this hypothesis unlikely, at least concerning C2C12 myotubes exposed to TNFα. In C2C12 differentiated myocytes, hyperexpression of follistatin, a physiological inhibitor of myostatin, indeed rescues TNFα-induced alterations in terms both of myotube size and of MyHC content. Nevertheless, although the effects of follistatin hyperexpression are phenotypically comparable to those exerted by ERK inhibition, the mechanisms involved are quite different. While PD restores myotube size and MyHC content by blocking ERK without affecting Akt activity, the opposite occurs in follistatin-hyperexpressing cultures, wherein ERK activation remains comparable to non-transfected TNFα-treated myotubes and p-Akt levels markedly increase. This latter observation is in line with previous reports showing that myostatin can lead to inhibition of the PI3K/Akt pathway [40] and, conversely, myostatin blockade results in increased p-Akt levels [41].

### ERK impinges on myocyte differentiation both *in vivo* and *in vitro*

The improved muscle trophism resulting from ERK inhibition appears achieved, at least in part, by prevention of atrogin-1 hyperexpression that likely depends on proinflammatory cytokines. However, the increase in atrogin-1 expression, although significant, is not really prominent, suggesting that other mechanisms are likely to contribute to muscle wasting in the C26 hosts.

Among the targets of ERK is the AP-1 transcription factor, which is activated in tumor-bearing animals [20] and may contribute to muscle atrophy, since this latter is improved inhibiting AP-1 by a c-jun dominant negative (TAM67) [42]. Little is known about the role that genes regulated by AP-1 may play in muscle depletion. An intriguing hypothesis is that, by inducing cyclin D1 expression [42], AP-1 could stimulate satellite cell proliferation not followed by differentiation, however, resulting in impaired myogenesis. Satellite cell phenotype is defined by the differential expression of specific factors, among which Pax7, MyoD and myogenin. While MyoD remains generally detectable, although at variable levels, high and low expression of Pax7 and myogenin, respectively, characterizes proliferating satellite cells, while the opposite pattern defines differentiating cells [43]. The relevance of TNFα to the regulation of the myogenic program is demonstrated by studies showing that increased cytokine levels inhibit skeletal myogenesis both *in vivo* and *in vitro* [5], [44], [45].

Satellite cell activation and differentiation appear required for the maintenance of skeletal muscle mass [46]. Muscle atrophy induced in mice by aging or hindlimb suspension is associated with loss of muscle precursor cells, resulting in reduced regenerative potential [19]. Recent studies reported that the skeletal muscle of tumor-bearing mice is markedly infiltrated by bone marrow-derived stem cells, suggesting the occurrence of a compensatory mechanism aimed at counteracting the wasting stimuli [47]. Alterations of the myogenic process were proposed to play a determinant role in the pathogenesis of muscle atrophy [5]. Normal mice exposed to TNFα and IFNγ combined developed muscle hypotrophy, associated with decreased MyoD levels, through a mechanism involving NF-κB [5]. Reduced MyoD levels, but unchanged NF-κB DNA-binding, were detected in a TNFα-dependent experimental model of cancer cachexia [20]. Finally, TNFα was proposed to abrogate satellite cell function, thereby delaying or inhibiting mice muscle regeneration after injury [48], and consistently, a reduced number of regenerating fibers was observed in TNFα-hyperexpressing muscles [45].

The present study demonstrates that in the muscle of the C26 hosts Pax7 expression is significantly increased with respect to controls, while myogenin levels are reduced. In previous reports Pax7 overexpression was found to result in inhibition of myogenesis [49]. In this regard, the pattern exhibited by Pax7 and myogenin in the skeletal muscle of C26 hosts is plainly compatible with an impaired regenerative process and suggests the possibility that activated satellite cells accumulate in tumor host muscle because of either enhanced proliferation or impaired differentiation or both. Altered expression of myogenic factors was previously reported in AH-130 hepatoma-bearing rats [20], in cancer patients [50], and in an experimental model of chronic kidney disease [51]. In the latter report, downregulation of IGF-1 signaling appeared to impair regeneration [51]. The present study suggests an alternative mechanism based on ERK activation: when the C26 hosts are treated with PD, and ERK is thus inhibited, Pax7 and myogenin expression is restored to control values. These observations suggest that ERK activation likely contributes to maintain satellite cells in an undifferentiated state.

The involvement of ERK in impairing the myogenic program in the muscle of cachectic animals was investigated in details in differentiating C2C12 myocytes. During differentiation, phosphorylated ERK, occurring at high levels over days 1–3, progressively declines concomitantly with Pax7 reduction and myogenin and MyoD increases (Fig. S5) [52]. In day 2-differentiating myocytes exposed to TNFα MyoD and myogenin decrease, but significantly increase on ERK inhibition. A different pattern can be observed for Pax7, whose expression in both differentiating myoblasts (Fig. S5) and terminally differentiated myotubes is virtually abrogated by TNFα (not shown), this change being only partially prevented by PD administration. This observation apparently is in contrast with the pattern shown by Pax7 in the C26 hosts. A possible explanation could reside in the multiple actions exerted by Pax7, which not only is required to promote the proliferation of undifferentiated cells, but is also necessary for the myogenic differentiation to proceed [53]. ERK-dependent reduction in Pax7 expression was also shown in differentiating C2C12 myoblasts treated with myostatin [52].

### Conclusions

The results shown in the present study demonstrate that ERK plays a crucial role in the pathogenesis of muscle wasting in cancer cachexia. ERK activation results in: 1) hyperexpression of the muscle-specific ubiquitin ligase atrogin-1, suggesting that an upregulation of UPS activity eventually supports protein hypercatabolism; 2) downregulation of the myogenic process, characterized by accumulation of activated satellite cells not competent to proceed into the differentiative program. In the adult tissue, the regenerative program is a normal response to an antecedent injury, yet the frame of reference for the modulations of myogenesis in cancer cachexia is poorly defined. In this regard, a previous study reported the occurrence of sarcoplasmic membrane leakage in mice bearing the C26 tumor [54]. Another possibility is that myogenesis is activated to compensate for a loss of myonuclei [47]. Irrespective of the stimulus leading to muscle regeneration, however, the observation that ERK inhibition can rescue satellite cells towards differentiation offers new clues for a better understanding of the pathogenetic mechanisms of muscle depletion in cancer cachexia and should be extensively explored in order to outline molecular targets potentially relevant to its treatment.

## Supporting Information

Figure S1 PD98059 administration (3 mg/kg) counteracts the onset of cachexia in C26-bearing mice. A) Body weight changes (i.b.w. of C = 18.93±1.38 g; C26 = 18.07±1.06 g; PD = 18.24±1.75 g; C26 PD = 17.96±1.65 g), (B) muscle weight in controls (n = 6) and C26 hosts (n = 8) either untreated or administered PD (3 mg/kg). Data (means ± SD) are expressed as percentages of controls. Significance of the differences: *p<0.05 vs C; $ p<0.05 vs C26.
(0.24 MB TIF)

Figure S2 PD98059 administration (3 mg/kg) counteracts the onset of cachexia in AH-130-bearing rats. A) Body weight changes, (B) muscle weight in controls (n = 6) and AH-130 hosts (n = 8) either untreated or administered PD (3 mg/kg, s.c.). Data (means ± SD) are expressed as percentages of controls. Significance of the differences: *p<0.05 vs C; **p<0.01 vs C; $ p<0.05 vs AH-130.
(0.25 MB TIF)

Figure S3 PD98059 administration does not affect circulating IL-6 levels in TB mice. IL-6 plasma levels (pg/ml) of TB mice (n = 8) and controls (n = 6), treated or not with PD (1 mg/kg), expressed as percentage of controls. Data are means ± SD. Significance of the differences: **p<0.01 vs C.
(0.19 MB TIF)

Figure S4 Myogenin is hyperphosphorylated in the GSN of C26-bearing mice. Myogenin expression assayed in C (n = 2) and TB mice (n = 2) on 30 µg of GSN cytosolic proteins, incubated (30′, 37°C) in 2 mM MnCl2, 50 mM HEPES pH 7.5, 0.1 mM EGTA, 5 mM DTT, in the presence or in the absence of lambda phosphatase (PPase; 400 U), and heat-denaturated in sample-loading buffer. Western blotting conducted as described in Materials and Methods.
(0.29 MB TIF)

Figure S5 Myogenic differentiation in C2C12 cells is associated with ERK downregulation. p-ERK, MyHC, MyoD, myogenin and Pax7 protein levels assayed on protein lysates from growing myoblasts (‘myobl’) or cells differentiated for 1 to 6 days. Densitometric quantifications were normalized according to tubulin levels. Data (means ± SD; n = 2) are expressed as percentages of growing myoblasts.
(0.45 MB TIF)
