# Supplementary figures and images for: Muscle Wasting and Impaired Myogenesis in Tumor Bearing Mice Are Prevented by ERK Inhibition
Source: PLoS One. 2010 Oct 27;5(10):e13604. doi: 10.1371/journal.pone.0013604 (PMC2965098; doi:10.1371/journal.pone.0013604)

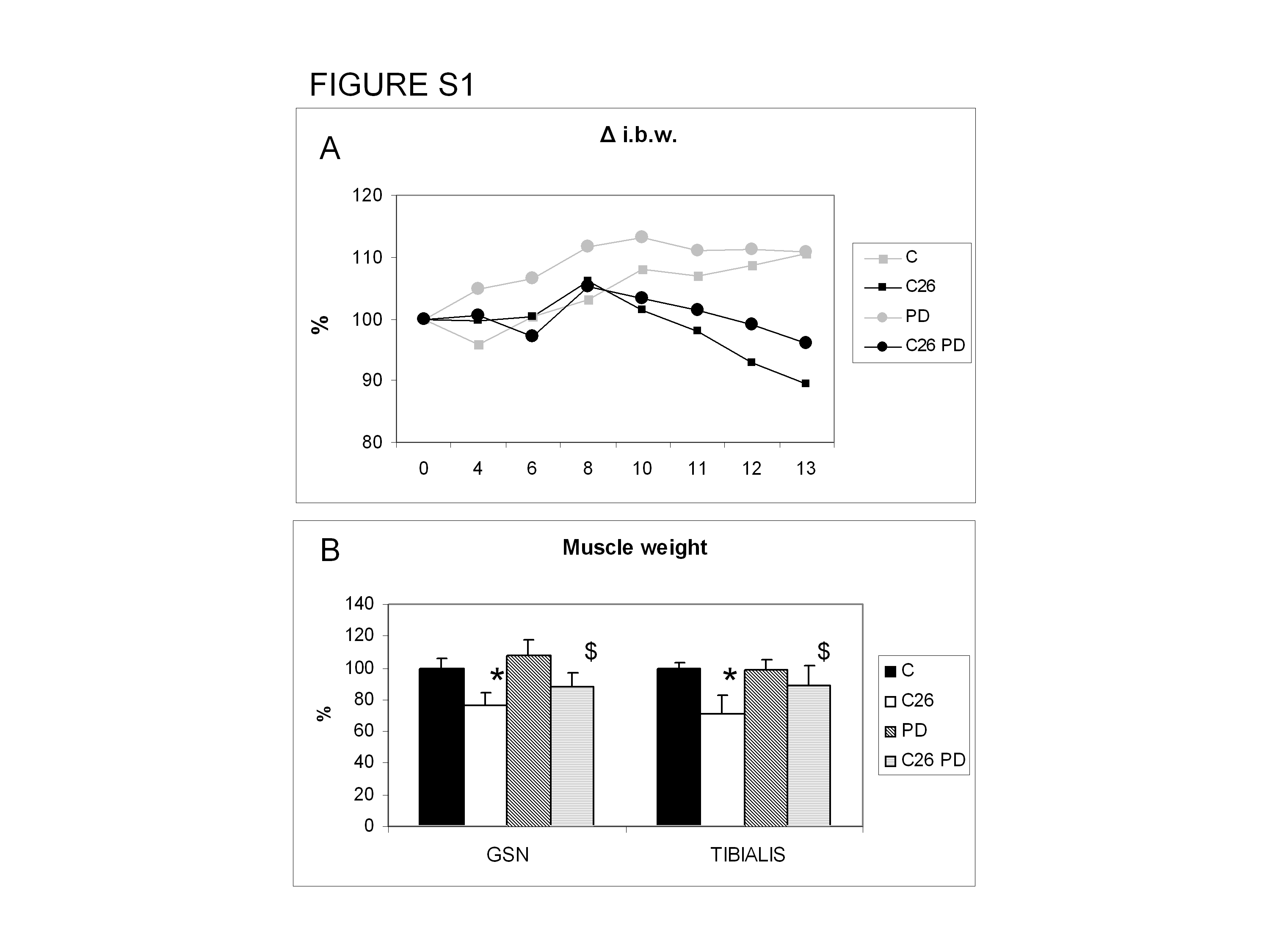

Supplement: Figure S1 — PD98059 administration (3 mg/kg) counteracts the onset of cachexia in C26-bearing mice. A) Body weight changes (i.b.w. of C = 18.93±1.38 g; C26 = 18.07±1.06 g; PD = 18.24±1.75 g; C26 PD = 17.96±1.65 g), (B) muscle weight in controls (n = 6) and C26 hosts (n = 8) either untreated or administered PD (3 mg/kg). Data (means ± SD) are expressed as percentages of controls. Significance of the differences: *p<0.05 vs C; $ p<0.05 vs C26. (0.24 MB TIF) [file pone.0013604.s001.tif]

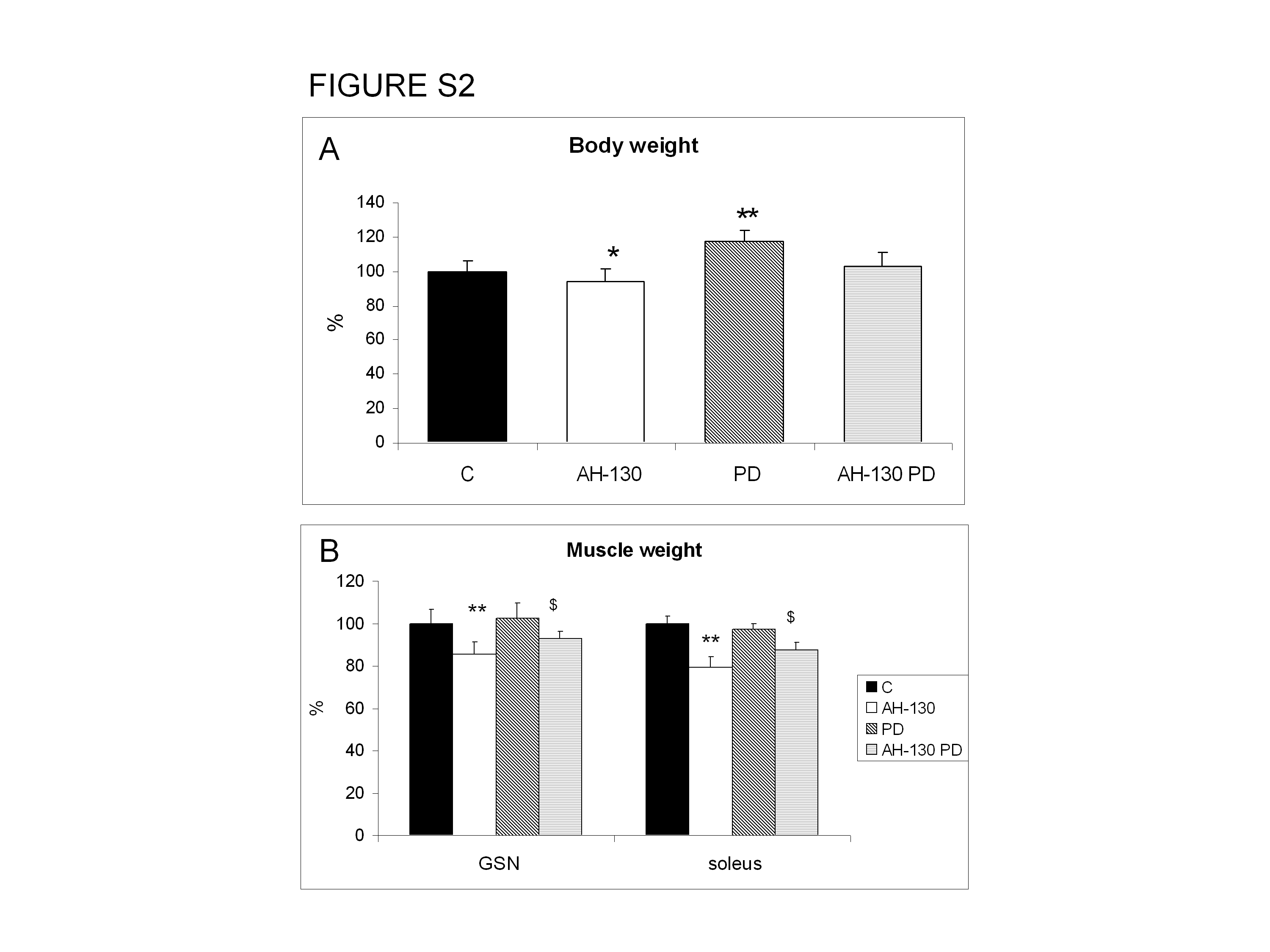

Supplement: Figure S2 — PD98059 administration (3 mg/kg) counteracts the onset of cachexia in AH-130-bearing rats. A) Body weight changes, (B) muscle weight in controls (n = 6) and AH-130 hosts (n = 8) either untreated or administered PD (3 mg/kg, s.c.). Data (means ± SD) are expressed as percentages of controls. Significance of the differences: *p<0.05 vs C; **p<0.01 vs C; $ p<0.05 vs AH-130. (0.25 MB TIF) [file pone.0013604.s002.tif]

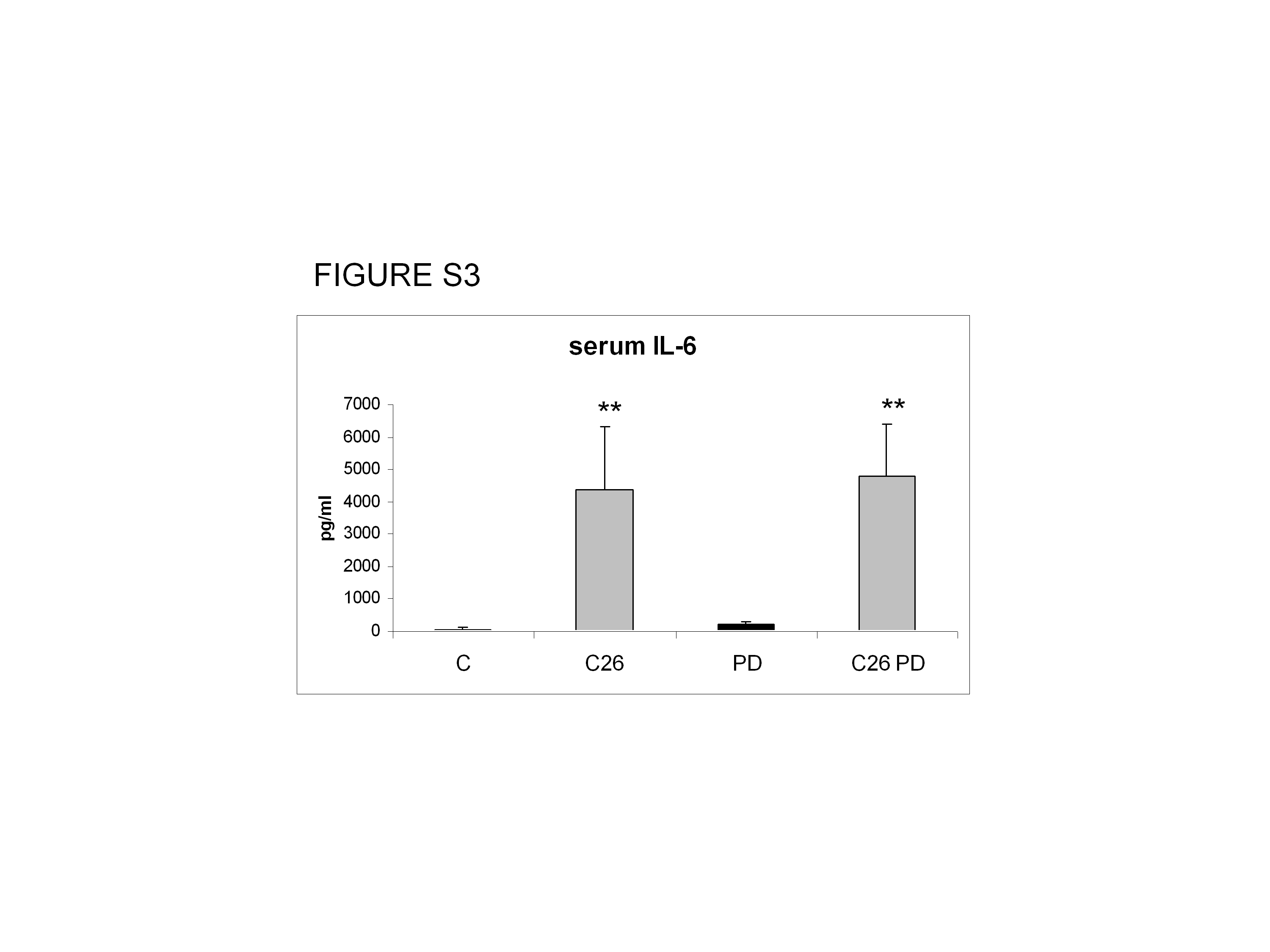

Supplement: Figure S3 — PD98059 administration does not affect circulating IL-6 levels in TB mice. IL-6 plasma levels (pg/ml) of TB mice (n = 8) and controls (n = 6), treated or not with PD (1 mg/kg), expressed as percentage of controls. Data are means ± SD. Significance of the differences: **p<0.01 vs C. (0.19 MB TIF) [file pone.0013604.s003.tif]

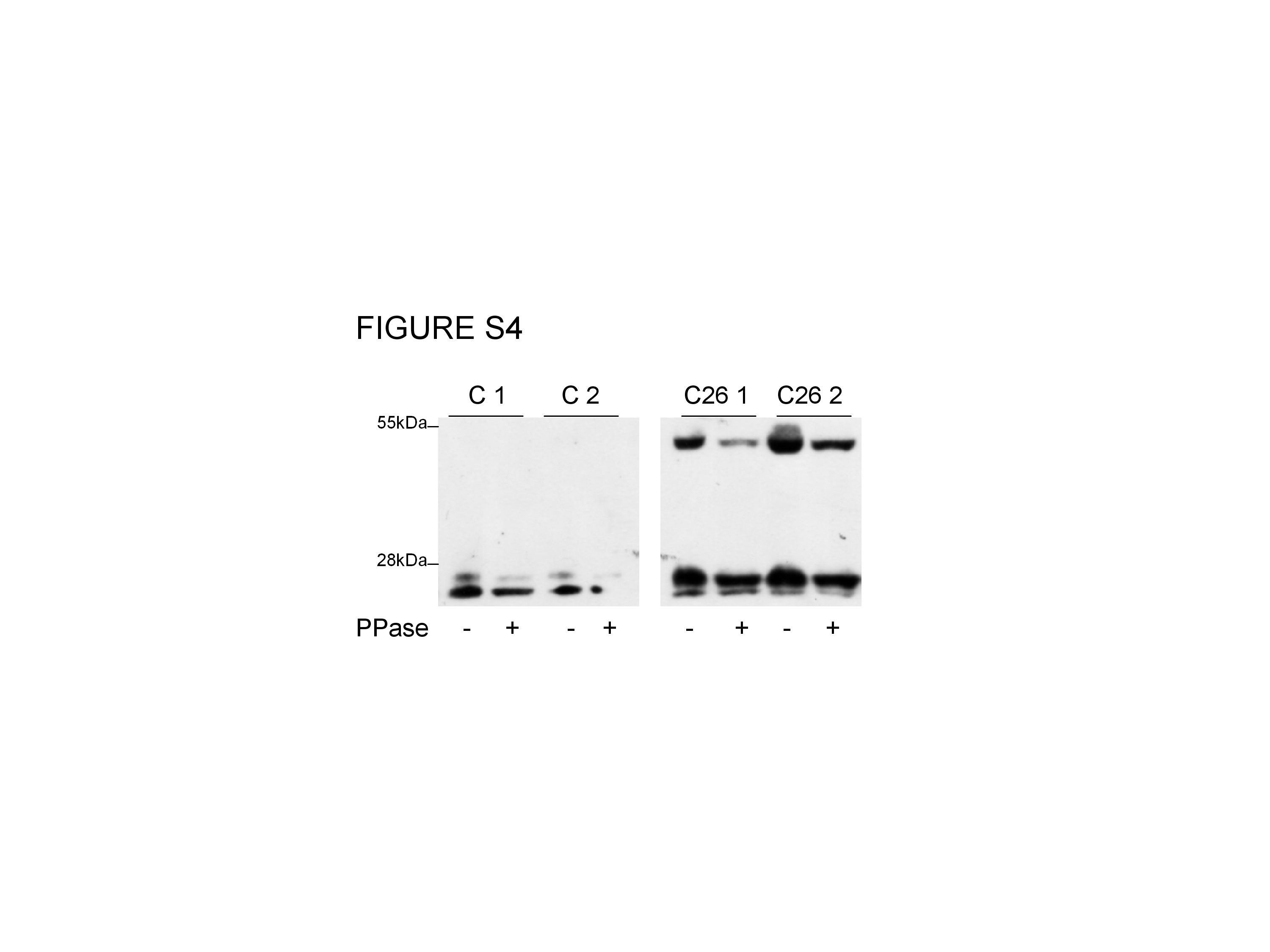

Supplement: Figure S4 — Myogenin is hyperphosphorylated in the GSN of C26-bearing mice. Myogenin expression assayed in C (n = 2) and TB mice (n = 2) on 30 µg of GSN cytosolic proteins, incubated (30′, 37°C) in 2 mM MnCl2, 50 mM HEPES pH 7.5, 0.1 mM EGTA, 5 mM DTT, in the presence or in the absence of lambda phosphatase (PPase; 400 U), and heat-denaturated in sample-loading buffer. Western blotting conducted as described in Materials and Methods. (0.29 MB TIF) [file pone.0013604.s004.tif]

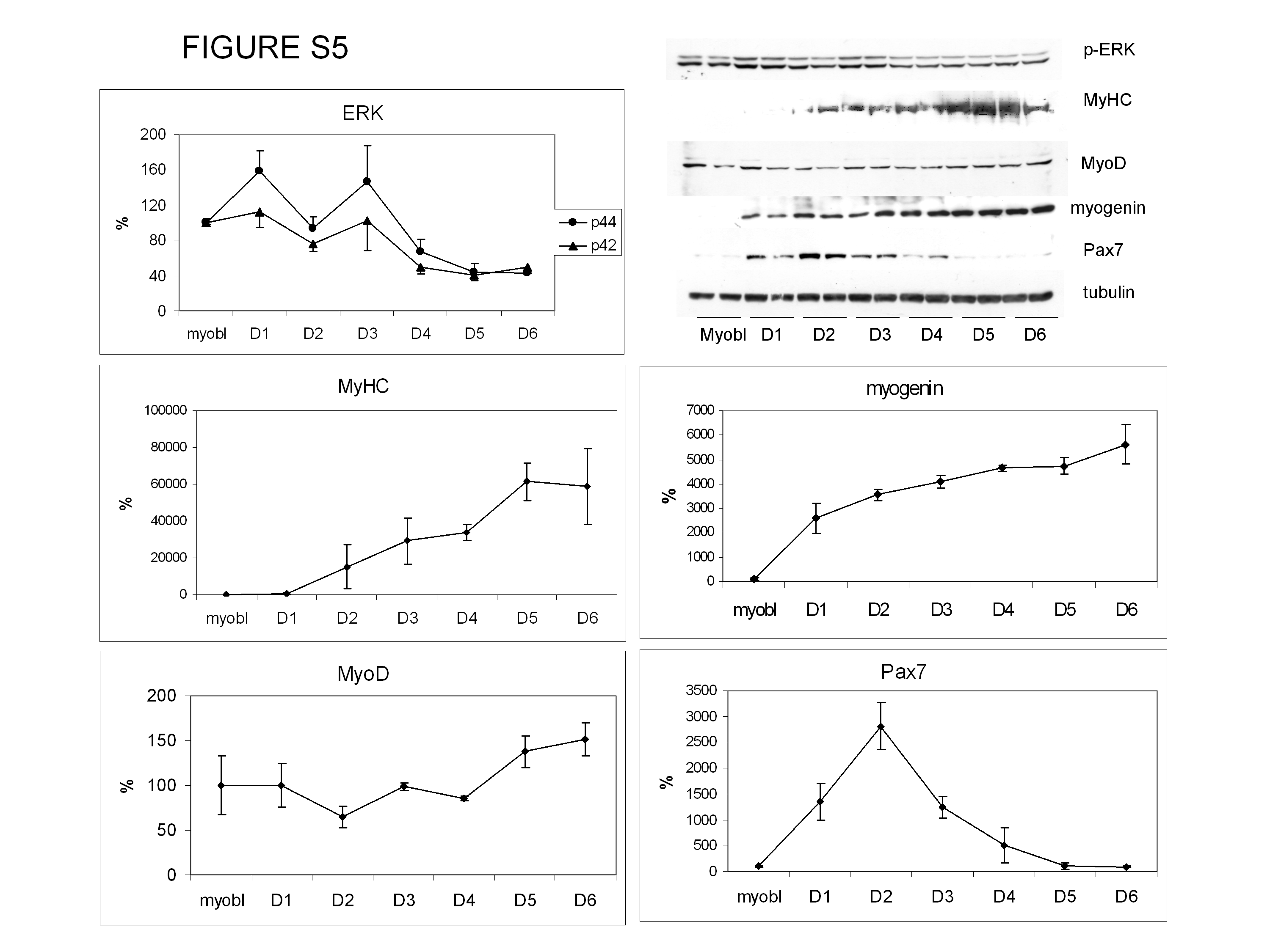

Supplement: Figure S5 — Myogenic differentiation in C2C12 cells is associated with ERK downregulation. p-ERK, MyHC, MyoD, myogenin and Pax7 protein levels assayed on protein lysates from growing myoblasts (‘myobl’) or cells differentiated for 1 to 6 days. Densitometric quantifications were normalized according to tubulin levels. Data (means ± SD; n = 2) are expressed as percentages of growing myoblasts. (0.45 MB TIF) [file pone.0013604.s005.tif]
